# Theranostic Protein Targeting ErbB2 for Bioluminescence Imaging and Therapy for Cancer

**DOI:** 10.1371/journal.pone.0075288

**Published:** 2013-09-17

**Authors:** Xiao-Jian Han, Ling-Fei Sun, Yuki Nishiyama, Bin Feng, Hiroyuki Michiue, Masaharu Seno, Hideki Matsui, Kazuhito Tomizawa

**Affiliations:** 1 Department of Physiology, Okayama University Graduate School of Medicine, Dentistry and Pharmaceutical Sciences, Okayama, Japan; 2 Department of Molecular Physiology, Faculty of Life Sciences, Kumamoto University, Kumamoto, Japan; 3 Japan Society for the Promotion of Science, Tokyo, Japan; 4 Department of Medical and Bioengineering Science, Graduate School of Natural Science and Technology, Okayama University, Okayama, Japan; 5 Institute of Translational Medicine, Nanchang University, Nanchang, China; 6 Department of Biotechnology, Dalian medical University, Dalian, China; NIH, United States of America

## Abstract

A combination of molecular-targeted cancer imaging and therapy is an emerging strategy to improve cancer diagnosis and minimize the side effects of conventional treatments. Here, we generated a recombinant protein, EC1-GLuc-p53C, by fusing EC1 peptide, an artificial ligand of ErbB2, with *Gaussia* luciferase (GLuc) and a p53-activating peptide, p53C. EC1-GLuc-p53C was expressed and purified from *E. coli* BL21. *In vitro* experiments showed that EC1-GLuc-p53c was stable in luminescent activity and selectively targeted ErbB2-overexpressing BT474 cells for bioluminescence imaging. Moreover, the internalized EC1-GLuc-p53C in BT474 cells exerted its function to reactivate p53 and significantly inhibited cellular proliferation. In tumor-bearing mice, the ErbB2-targeted bioluminescence imaging and therapeutic effect of EC1-GLuc-p53C were also observed specifically in BT474 tumors but not in MCF7 tumors, which does not overexpress ErbB2. Thus, the present study demonstrates EC1-GLuc-p53C to be an effective theranostic reagent targeting ErbB2 for bioluminescence imaging and cancer therapy.

## Introduction

ErbB2 is a member of the epidermal growth factor receptor (EGFR) family of receptor tyrosine kinases, also called the ErbB family. The ErbB family includes four members, ErbB1, ErbB2, ErbB3 and ErbB4. There are several endogenous ligands for ErbB receptors with the exception of ErbB2 [1]. The tyrosine kinase activity of ErbBs can be activated by endogenous ligands and the homo- or hetero-dimerization of ErbB receptors, which is involved in the regulation of cellular proliferation and cell survival [1-3]. In addition, ErbB2 has been implicated in tumor pathogenesis and progression [4-6]. Clinically, overexpression of ErbB2 is associated with approximately 30% of breast cancers, ovarian cancers [7] and other common types of cancers including lung, gastric, and oral cancers [8]. The overexpression is also associated with the metastasis, therapeutic resistance and poor prognosis of cancer [9-11]. Thus, ErbB2 may be a promising molecular target for cancer imaging and treatment using monoclonal antibodies and peptide-targeting vectors [12,13]. In a phage display study, several small artificial cyclic peptides with specific affinity for ErbB2 were identified. EC1, one of these artificial peptides, bound the extracellular domain of ErbB2 in living cells and fresh frozen human breast cancer specimens [14]. Moreover, biotin-conjugated EC1 and the recombinant protein EC1-eGFP retained affinity for ErbB2 and were internalized by ErbB2-overexpressing cancer cells [14,15]. Recently, divalent and multivalent forms of EC1-Fc ligand in liposomes were reported to improve affinity for ErbB2 and enhance internalization [16]. Thus, EC1 peptide may be a potential artificial ligand for targeting ErbB2.

In tumor pathogenesis, several abnormal mutations are found in tumor-suppressor genes. One of the best-known tumor-suppressor genes is *p53*, which is an important regulator of apoptosis and the most commonly mutated gene in cancer [17,18]. Restoration of p53 activity has been proved to be an effective means of treating cancer cells with p53 mutations [19,20]. In our previous studies [21-23], a full-length recombinant p53 was successfully delivered into human malignant glioma cells, oral and bladder cancer cells by fusing with poly-arginine, a cell-penetrating peptide (CPP). It was found that the full-length recombinant p53 had a significant inhibitory effect on the proliferation of cancer cells. However, the large molecular size and rapid degradation by the ubiquitin-proteasome pathway greatly influenced the efficiency of protein transduction and effect of the transduced p53 on cancer cells [24]. The C-terminus of p53 is a lysine-rich domain subject to a variety of posttranslational modifications [25,26]. A peptide derived from the C-terminus activates specific DNA binding with p53 *in vitro* through an unknown mechanism [27]. It was reported that the p53-derived C-terminal peptide (p53C) induced rapid apoptosis in breast cancer cells carrying endogenous p53 mutations or overexpressed wild-type (wt) p53, but was not toxic to nonmalignant human cell lines containing wt p53 [28]. In addition, p53C peptide fused with CPP inhibits the proliferation of cancer cells by reactivating endogenous p53, and significantly increases lifespan in animal models of terminal peritoneal carcinomatosis and bladder cancer *in vivo* [29-31]. These studies demonstrate the reactivation of the p53 protein by p53C peptide to be a promising means for cancer therapy.

Bioluminescence imaging is emerging as a relatively simple, cost-effective and extremely sensitive way to monitor dynamic biological processes in intact cells and living animals [32]. In recent years, the technology has developed rapidly with improvements in luciferase reporters and instrumentation [33]. The most common luciferases for bioluminescence imaging include *Firefly* luciferase (FLuc), *Renilla* luciferase (RLuc) and *Gaussia* luciferase (GLuc). Each luciferase has distinct properties in the application of bioluminescence imaging. FLuc (62 kDa) catalyzes the oxidation of luciferin to yield bioluminescence in the presence of O_2_, magnesium and ATP [34]. RLuc (36 kDa) and GLuc (19.9 kDa) catalyze the oxidative decarboxylation of coelenterazine to emit light independent of ATP. However, RLuc has a lower quantum yield than FLuc, and also less enzymatic efficiency [35,36]. GLuc yields approximately 200- (*in vivo*) to 1000-fold (*in vitro*) more bioluminescence in mammalian cells than FLuc and RLuc [37]. Therefore, its small molecular size (19.9kDa), independence from ATP and strong emission make GLuc much more suitable for bioluminescence imaging *in vitro* and *in vivo* [38,39].

The concept of “Theranostic” was originated by Funkhouser in 2002 from one of his reviews [40]. Theranostics is defined as a material that combines the modalities of therapy and diagnostic imaging at the same time within the same dose. The goal of theranostic is to donate materials with the capacity of monitoring the treated tissue and efficacy in the long-term period [41]. Theranostic reagents have been developed fast in the previous decade, especially after the emergence of some new optical probes and potent biomolecules for targeting and therapy. In the present study, a novel ErbB2-targeting bioluminescence protein was constructed by fusing EC1 with GLuc and p53C peptide. Two human breast carcinoma cell lines, MCF7 without expression of ErbB2 and BT474 overexpressing ErbB2, were employed to evaluate the role of EC1-GLuc-p53C in specific bioluminescence imaging and cancer therapy. We observed that EC1-GLuc-p53C not only targeted ErbB2 for bioluminescence imaging, but also selectively inhibited cell proliferation and tumor growth of BT474 *in vitro* and *in vivo*. The present results indicate that EC1-GLuc-p53C may be a promising theranostic reagent targeting ErbB2 for bioluminescence imaging and therapy.

## Materials and Methods

### Cell culture and detection of ErbB2 expression

The MCF7 and BT474 cell lines were obtained from the American Type Culture Collection (ATCC). BT474 cells carry a missense mutation (E285K) in the p53 gene [42], while MCF7 cells with overexpression of wt p53 [28]. BT474 cells were grown in a RPMI-1640 medium supplemented with 10% fetal bovine serum (FBS) and 100 µg/ml penicillin-streptomycin (P/S). MCF7 cells were cultured in DMED medium supplemented with 10% FBS and 100 µg/ml P/S. The mediums, FBS and P/S were from Invitrogen. All cultures were maintained in a humidified incubator at 37°C with an atmosphere containing 5% CO_2_.

The expression of ErbB2 in MCF7 and BT474 cells was examined by Western immunoblotting. Briefly, MCF7 and BT474 cells on 35mm dishes were washed once with PBS, and scraped in 1×SDS sample buffer. Cell lysates were subjected to 6% SDS-PAGE, and immunoblotted with Rabbit anti-ErbB2 antibody (1:1000; Cell Signaling) overnight at 4°C. HRP-conjugated secondary antibody (Sigma-Aldrich) was used at 1:2000. Immunoblotting signals were detected by the Versa Doc 5000 imaging system (Bio-Rad) with an enhanced chemiluminescence detection kit (Amersham Biosciences, Pittsburgh, PA).

### Plasmid construction

The pGLuc plasmid was purchased from LUX biotechnology Ltd (Scotland, UK). The GLuc gene was amplified from the plasmid using a sense primer (5'- GGATCCGAAACCAACTGAAAACAATGAAG-3') and antisense primer (5'-GTCGACATCACCACCGGCACCCTTTAT-3'). The PCR product was ligated into the pCR2.1 TOPO vector (Invitrogen) for amplification, and recloned in the pET52b vector (Invitrogen) to construct GLuc-pET52b. The following sense and antisense oligonucleotides (Hokkaido System Science, Japan) with appropriate resistance enzyme sites for EC1 or p53C peptide were prepared; for EC1; 5'-GGGTGGACTGGCTGGTGCCTGAATCCAGAAGAATCTACTTGGGGATTCTGTACTGGATCTTTCGGTGGAGGTAGTTCAG-3' (sense, underline indicates *Sam* I and *Bam*HI sites) and 5'-GATCCTGAACTACCTCCACCGAAAGATCCAGTACAGAATCCCCAAGTAGATTCTTCTGGATTCAGGCACCAGCCAGTCCACCC-3' (antisense, underline indicates *Bam*HI and *Sam* I sites), and for p53C; 5'- TCGACGGGAGCAGGGCTCACTCCAGCCACCTGAAGTCCAAAAAGGGTCAGTCTACCTCCCGCCATAAAAAAGGCGC-3' (sense, underline indicates *Sal*Ⅰand *Not*Ⅰsites), and 5'-GGCCGCGCCTTTTTTATGGCGGGAHHTAGACTGACCCTTTTTGGACTTCAGGTGGCTGGAGTGAGCCCTGCTCCCG-3' (sense, underline indicates *Not*Ⅰand *Sal*Ⅰsites). The oligonucleotides for EC1 and p53C were modified by phosphorylation at 5'. The sense and antisense oligonucleotides (10 pmol each) were co-incubated at 95°C for 10 min, and annealed at room temperature to form double-stranded DNA. The double-stranded DNA for EC1 or p53C was ligated to GLuc-pET52b treated with the appropriate resistance enzymes to construct EC1-GLuc-pET52b or EC1-GLuc-p53C-pET52b. Site-directed mutagenesis was introduced at BamHI in EC1-GLuc and EC1-GLuc-p53C using a QuikChange kit (Stratagene) according to the manufacturer’s instructions. The primers for mutagenesis were 5'-GTGGAGGTAGTTCAGGAACCAAACCAACTG-3' (sense, underline indicates the mutated nucleotide), 5'-CAGTTGGTTTGGTTCCTGAACTACCTCCAC-3' (antisense). GLuc, EC1-GLuc and EC1-GLuc-p53C were then subcloned into pGEX-6p-1 between the *Bam*HI and *Eco*RI sites for protein purification. The sequences of constructed plasmids were confirmed using an ABI 3100 sequencer.

### Expression and purification of recombinant proteins

The expression and purification of recombinant proteins were performed as described previously [21]. Briefly, *E. coli* BL21 (DE3) transformed with the plasmid for GLuc, EC1-GLuc or EC1-GLuc-p53C was grown in a LB medium containing 100 µg/mL ampicillin at 37°C. When the OD600 reached 0.6, expression of the GST-fused proteins was induced by adding 0.2 mM isopropyl 1-thio-β-D-galactopyranoside at 25°C overnight. The fusion proteins were purified using a column of glutathione Sepharose 4 Fast Flow (GE Healthcare). To cleave GST from the fusion proteins, the purified proteins were further treated with PreScission protease (GE Healthcare) according to the manufacturer’s instructions. Recombinant proteins were subjected to 15% SDS-PAGE, and confirmed by Coomassie brilliant blue staining and immunoblotting with rabbit anti- *Gaussia* Luciferase serum (1:1000, Nanolight). The proteins were finally dialyzed against PBS, and concentrations were determined using a protein assay kit (Bio-Rad Laboratories). Aliquots of protein were stored at -80°C prior to use.

### Preparation of coelenterazine (CTZ)

The substrate for GLuc was prepared as described previously [32]. Briefly, CTZ (Nanolight) was dissolved in acidified methanol (1 drop of concentrated HCl (12.4 N) in 10 mL of methanol) to a concentration of 5mg/ml. Aliquots of 100µl were stored at -80°C. For luminescence imaging, the aliquots of CTZ (5mg/mL) were diluted with PBS (containing 5mM NaCl, pH 7.2) for higher light output and more stability.

### Assays of the luminescent activity and stability of recombinant proteins

To determine the luminescent activity of the purified proteins, 2µg of each protein was transferred to a 96-well plate. Bioluminescence was measured using a plate luminometer (Microlumat plus LB 96V, Berthold technologies) after the addition of 10 µl of CTZ (20 µM). Bioluminescent activity was reported in relative light units (RLU) measured with a 10 sec integration time and calculated as RLU per micromole for each protein. The values for EC1-GLuc and EC1-GLuc-p53C were normalized with GLuc.

The stability of recombinant proteins was assessed as described previously [38]. Aliquots of protein were incubated with an equal volume of mouse serum (Sigma) at 37°C. At each time point, samples were withdrawn for bioluminescence imaging and Western blotting. In the analysis of bioluminescence stability, the percentage of the initial amount of RLU at each time point was plotted for each protein. To detect the degradation of proteins after the incubation with serum, samples withdrawn at each time point were subjected to electrophoresis in 15% acrylamide SDS-PAGE gel, and immunoblotted with rabbit anti- *Gaussia* Luciferase serum at a dilution of 1:1000. HRP-conjugated secondary antibody (Rabbit; Sigma-Aldrich) was used at 1:2000.

### Bioluminescence imaging *in vitro*, Internalization by ErbB2-overexpressing cells and WST-1 assay

For bioluminescence imaging *in vitro*, MCF7 and BT474 cells were cultured on 35-mm glass-bottomed dishes. To improve the adherence of MCF7 and BT474 cells, all glass-bottomed dishes were pre-coated with laminin (Roche). MCF7 and BT474 cells were incubated with 1µM of each protein for 24h. After three washes with DMEM, bioluminescence was detected with an Olympus Luminoview LV 200 (Bio-luminescence microscope) immediately after the addition of 1 µg/mL CTZ in serum-free DMEM. Images were acquired and analyzed with Metamorph software (Molecular devices).

To examine the internalization of EC1-fused proteins in ErbB2 overexpressing cancer cells, BT474 cells were incubated with 1 µM of GLuc, EC1-GLuc or EC1-GLuc-p53C. In blocking study, BT474 cells were treated with anti-ErbB2 antibody against extra-cellular domain of ErbB2 (Chicken, 1.0 µg/mL, abcam) 1 h prior to incubation with EC1-GLuc or EC1-GLuc-p53C. After 24 h, cells were washed with PBS three times and trypsinized. Cell lysate was prepared and subjected to 15% SDS-PAGE. The internalization of fusion protein was immunoblotted with rabbit anti- *Gaussia* Luciferase serum. β-actin was used as an endogenous control.

To evaluate the therapeutic effect of EC1-GLuc-p53C *in vitro*, cell viability was determined using a WST-1 assay as described previously [21]. After 3.0×10^3^ MCF7 and 1.0×10^4^ BT474 cells were seeded on 96-well flat-bottomed plates, they were cultured in DMEM or RPMI1640 medium containing 10% fetal bovine serum for 24 h. The cells were then supplemented with 1µM of protein or PBS (day0) and further cultured 96 h (day4). Cell viability from day 0 to day 4 was measured using the WST-1 assay according to the manufacturer’s instructions (Roche Applied Science). To determine the dose-dependent effect of EC1-GLuc-p53C on the proliferation of BT474 cells, the protein was used at 0.1 ~ 2.0 µM, and the WST-1 assay was conducted on day 4.

### Reporter Assay for p53-driven Transactivation

The reporter assay was performed as described previously [22]. Briefly, the luciferase reporter vector pGL2-basic (Promega) containing a 2.4 kbp fragment of the human p21^*WAF1*^ promoter was a gift from Drs. T. Akiyama (Tokyo University) and K. Yoshikawa (Osaka University). BT474 cells grown until 80% confluent in 35 mm-diameter dishes were transfected with the luciferase reporter vector by lipofectamine 2000 (Invitrogen). After 24 h, the cells were incubated with 1µM of GLuc, EC1-GLuc, EC1-GLuc-p53C or the same volume of PBS for 24h, and further cultured in new medium 2 h prior to cell harvest. The cell lysate was used for measuring luciferase activity with a luminometer and Luciferase Assay System (Promega). The background luciferase activity was subtracted in all experiments. Five independent experiments were performed for each condition.

### Preparation of tumor-bearing mice

Nude mice (Balb/c Slc-nu/nu, female, 6~8 weeks) were purchased from Charlesriver, Japan. The plan of animal experiments was reviewed and approved by the ethics committee for animal experiments of Okayama University under the IDs OKU-2010378, OKU-2011400. 17β-estradiol pellets (0.72 mg; Innovative Research of America) were implanted 4 days before tumor cell transplantation and remained in place until the end of the study. The cultured MCF7 and BT474 cells were washed twice with PBS, typsinized, and harvested by centrifuge. Cells (5.0×10^6^ cell) suspended in Matrigel (BD Bioscience) were transplanted subcutaneously on both flanks of anesthetized mice with intraperitoneally injection of 5mg/100g pentobarbital solution under aseptic conditions. Mice with tumors developed for 10~14 days were used for *in vivo* experiments. During tumor development, breath and behavioral activity were monitored twice every day to evaluate pain in mice. Experiments were immediately stopped if mice appeared significant symptom of pain. All mice were sacrificed by intraperitoneally injection of 15mg/100g pentobarbital solution after experiments.

The expression of ErbB2 in MCF7 and BT474 xenografts was confirmed by immunofluorescence (IF) staining. Paraffin-embedded slices of tumors were prepared at a thickness of 5 µm. First, histological observations of xenografted tumors were made using H&E staining. IF staining was then performed with anti-ErbB2 antibody (1:50; Cell Signaling). The secondary antibody was Cy3-conjugated anti-rabbit IgG (1:100; Invitrogen, Molecular probes). The nucleus was counter-stained with 0.1 µg/ml of Hoechst 33248 (Sigma) for 5 min. Fluorescence signals were observed using a confocal laser microscope (FV300, Olympus).

### Tumor site retention of EC1-GLuc-p53C in tumor xenografts

Next, 30 µL of EC1-GLuc-p53C protein (0.5 µg/µL) was injected intratumorally into MCF7 and BT474 xenografts in nude mice. At 1, 3, 6, 12 and 24 hours after the injection, the mice were sacrificed. Tumors were excised and immediately fixed with 4% paraformaldehyde. Paraffin-embedded slices of MCF7 and BT474 tumors were prepared at 10 µm. Immunofluorescence staining was carried out to analyze the distribution of EC1-GLuc-p53C in tumors. The immunofluorescence staining was performed according to the instructions that accompanied the rabbit anti- *Gaussia* Luciferase serum. The secondary antibody was FITC-conjugated rabbit IgG (1:100, Invitrogen, Molecular probes). Fluorescence signals were observed using a confocal laser microscope (FluoView, Olympus, Japan).

### Bioluminescence imaging *in vivo*

For bioluminescence imaging *in vivo*, 30 µl of EC1-GLuc-p53C protein (0.5 µg/µl) was intratumorally injected into the MCF7 and BT474 xenografts established in nude mice. At 6, 8, 12 and 24 hours after the injection, mice were imaged by injection via a tail vein with 100 µl of CTZ solution (3 mg/Kg) under anesthesia using a cooled CCD camera (IVIS Lumina-II, Caliper Life Sciences) as described previously [39]. The bioluminescent intensity of the selected region over the tumor was recorded as maximum photons s^-1^ cm^-2^ steradian^-1^.

### Effect of EC1-GLuc-p53C on tumor growth

MCF7 and BT474 xenografts on both flanks of nude mice were allowed to grow until an average volume of ~ 100mm^3^ ± 10mm^3^ was obtained prior to protein injection. Then, 30 µL of purified recombinant protein (0.5 µg/µL) or 30 µL of PBS was intratumorally injected into MCF7 and BT474 xenografts everyday for 5 days. The mice were monitored daily and their tumor volume was measured using digital vernier calipers twice week. During the whole course of therapy in tumor burden experiment no mouse died. Tumor volume was calculated according to the formula: tumor volume=L×W^2^×0.5 (L is the longest diameter, W is the shortest diameter).

### Statistical analysis

Data are shown as the mean±SD or mean±S.E.M. Data were analyzed using either Student’s t test to compare two conditions or ANOVA followed by planned comparisons of multiple conditions, and P<0.05 was considered to be significant.

## Results

### Purification and biochemical characterization of the recombinant proteins

**Figure 1:**
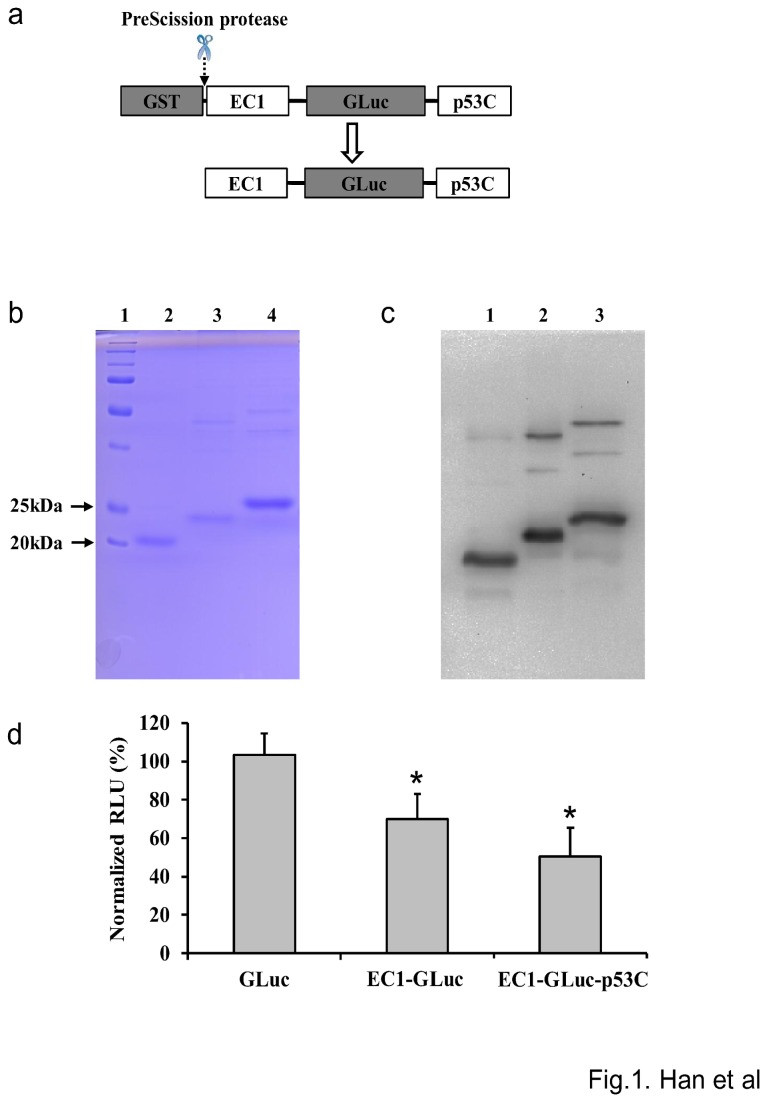
Purification and activity of the recombinant proteins. a) Schema of the purification of EC1-GLuc-p53C with the GST expression system. The GST tag was cleaved by PreScission protease after purification. b) Coomassie brilliant blue staining of purified proteins after 15% SDS-PAGE. Lane 1~4 are marker, GLuc, EC1-GLuc and EC1-GLuc-p53C, respectively. c) The purified proteins after 15% SDS-PAGE were detected using Western blotting with rabbit anti-*Gaussia* Luciferase serum. Lanes 1~3 are GLuc, EC1-GLuc and EC1-GLuc-p53C, respectively. d) Bioluminescent activities of the purified proteins. Each protein (2 µg) was evaluated using a plate luminometer. The bioluminescent values of EC1-GLuc and EC1-GLuc-p53C were normalized with GLuc. n=6 each, * p<0.01.

The three GST-fused protein constructs were expressed in *E. coli* BL21 and purified using a column of glutathione Sepharose 4 Fast Flow. After purification, the GST tag was cleaved by PreScission protease to harvest GLuc, EC1-GLuc and EC1-GLuc-p53C as shown in Figure 1a. GLuc and EC1-GLuc were used as control proteins. Coomassie brilliant blue staining revealed the purity of all three proteins to be > 80%. The molecular sizes of GLuc, EC1-GLuc and EC1-GLuc-p53C were approximately 20, 23, and 25KDa, respectively (Figure 1b). In Western blotting, the main bands of GLuc, EC1-GLuc and EC1-GLuc-p53C and additional minor bands corresponding to the GST-fused proteins were observed (Figure 1c). Moreover, a loss in bioluminescent activity was induced by the fusion of EC1 and p53C peptide with GLuc. An approximately 30% and 50% reduction was detected in the bioluminescent activity of EC1-GLuc and EC1-GLuc-p53C compared with GLuc, respectively (Figure 1d).

**Figure 2:**
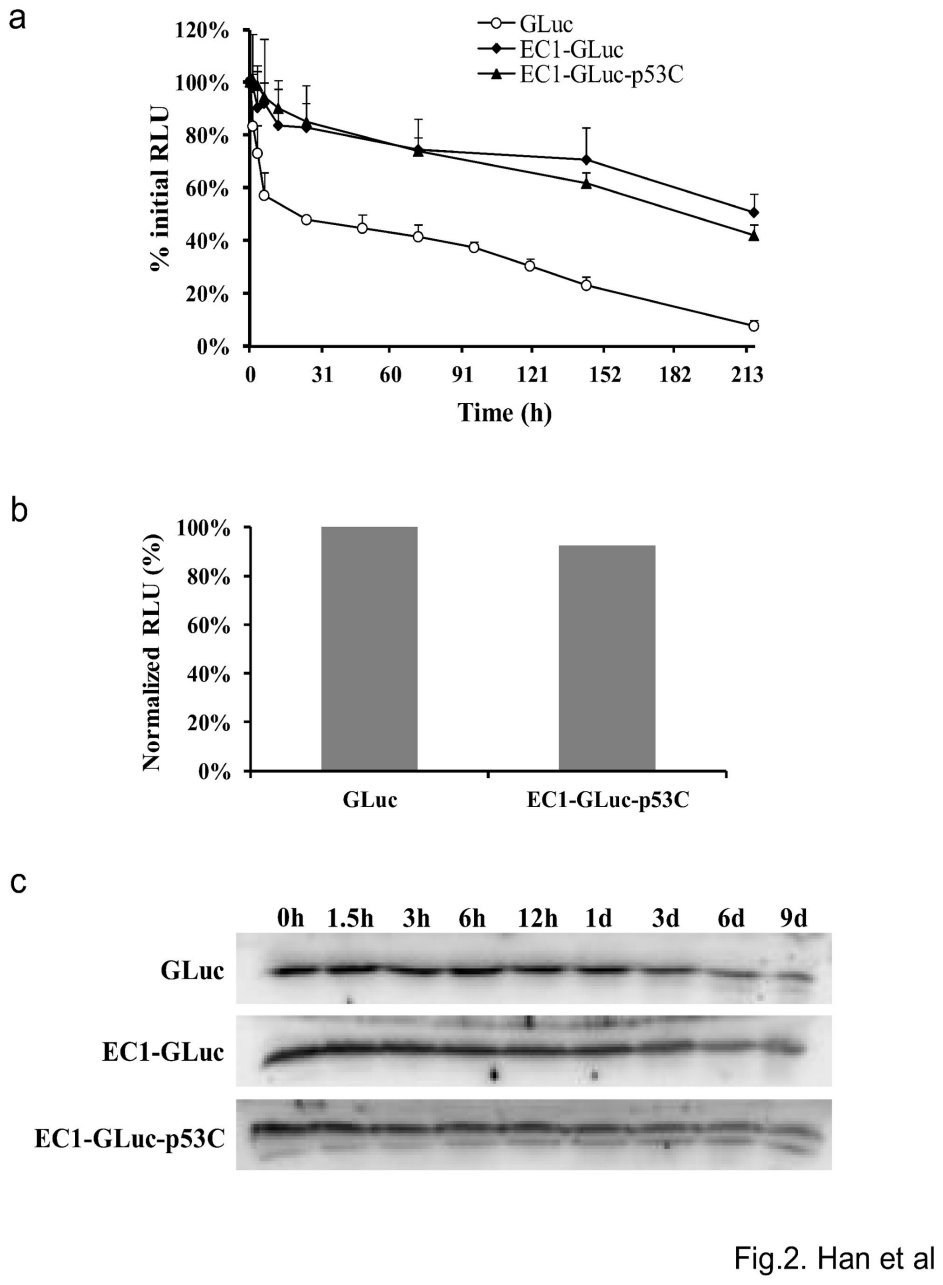
Stability and bioluminescent activity of the recombinant proteins in serum. Proteins incubated with an equal volume of mouse serum at 37°C were withdrawn at the indicated time points for bioluminescent activity evaluation (a) and Western blotting (c). b) The bioluminescent activity of EC1-GLuc-p53C and GLuc was examined 3 h after incubation in serum. The bioluminescent values of EC1-GLuc-p53C were normalized with GLuc. n = 6 each.

The stability of GLuc, EC1-GLuc and EC1-GLuc-p53C was evaluated by incubation in mouse serum at 37°C. The stability of GLuc was improved by the fusion of EC1 and p53C. The half-life of the luminescent activity of GLuc was determined as 18.6 hours, while that of EC1-GLuc and EC1-GLuc-p53C was 189.5 and 216 hours, respectively (Figure 2a). As a result, the bioluminescent activity of EC1-GLuc-p53C reached 92% that of GLuc at 3 h after incubation in serum (Figure 2b). In addition, the degradation of the three proteins in serum was also examined by Western blotting using anti-GLuc antibody. All three proteins were stable against degradation when incubated in serum at 37°C (Figure 2c). These results suggest the bioluminescent activity and stability of EC1-GLuc-p53C to be suitable for application both *in vitro* and *in vivo*.

### EC1-GLuc-p53C targets ErbB2 for bioluminescence imaging *in vitro*

**Figure 3:**
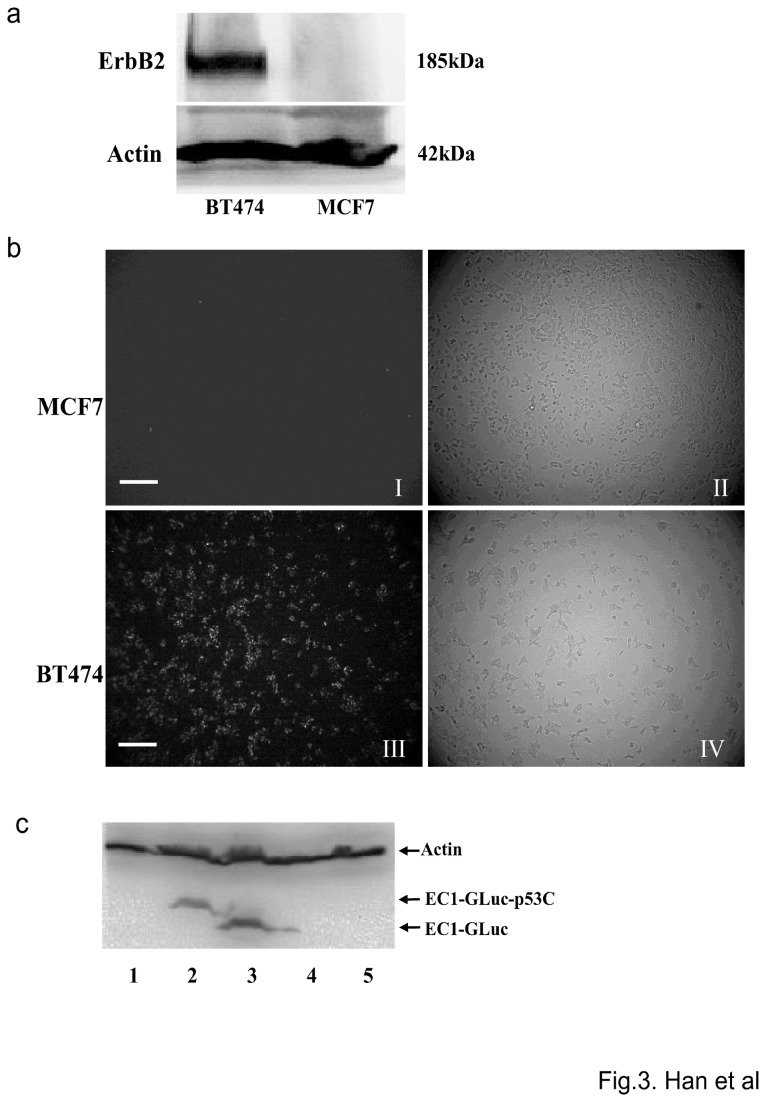
ErbB2-targeted bioluminescence imaging of EC1-GLuc-p53C *in vitro*. a) Expression of ErbB2 in MCF7 and BT474 cells. Cell lysate of the two cell lines was subjected to 6% SDS-PAGE and immunoblotted with anti-ErbB2 antibody. β-actin was used as an endogenous control. b) Bioluminescence imaging *in* *vitro*. Cells incubated with 1 µM of EC-GLuc-p53C were washed with culture medium without serum. Images were acquired with a bioluminescence microscope immediately after the addition of 1 µg/mL CTZ. I and III, bioluminescence images; II and IV, phase-contrast images. Bar = 100 µm. c) Internalization of EC1-fused proteins into ErbB2-overexpressing BT474 cells. Cells were incubated with 1 µM of protein for 24 h, lane 1: GLuc; lane 2: EC-GLuc-p53C; lane 3: EC-GLuc; lane 4: anti-ErbB2+EC1-GLuc-p53C; lane 5: anti-ErbB2+EC-GLuc. The internalization of fusion protein was immunoblotted with rabbit anti-*Gaussia* Luciferase serum. β-actin was used as an endogenous control.

Two human breast carcinoma cell lines, MCF7 and BT474, were used to evaluate the ErbB2-targeted imaging *in vitro*. The overexpression of ErbB2 in BT474 was confirmed by Western blotting, while the expression of ErbB2 in MCF7 cells was undetectable (Figure 3a). To detect the ErbB2-targeted bioluminescence, MCF7 and BT474 cells were treated with 1 µM of the recombinant proteins. A strong signal was detected in ErbB2-overexpressing BT474 cells incubated with EC1-GLuc and EC1-GLuc-p53C (Figure 3b, Figure S1). In contrast, no significant bioluminescence was observed in MCF7 cells incubated with each protein (Figure 3b, Figure S1). Moreover, internalization of the recombinant proteins was also observed in BT474 cells incubated with EC1-GLuc and EC1-GLuc-p53C, and the internalization of EC1-fused proteins was mostly undetectable, when the extra-cellular domain of ErbB2 was blocked by anti-ErbB2 antibody (Figure 3c). These results suggest that EC1-GLuc and EC1-GLuc-p53C retain affinity for ErbB2, and are internalized in ErbB2-overexpressing cells *in vitro*.

### EC1-GLuc-p53C selectively inhibits proliferation of ErbB2-overexpressing BT474 Cells

**Figure 4:**
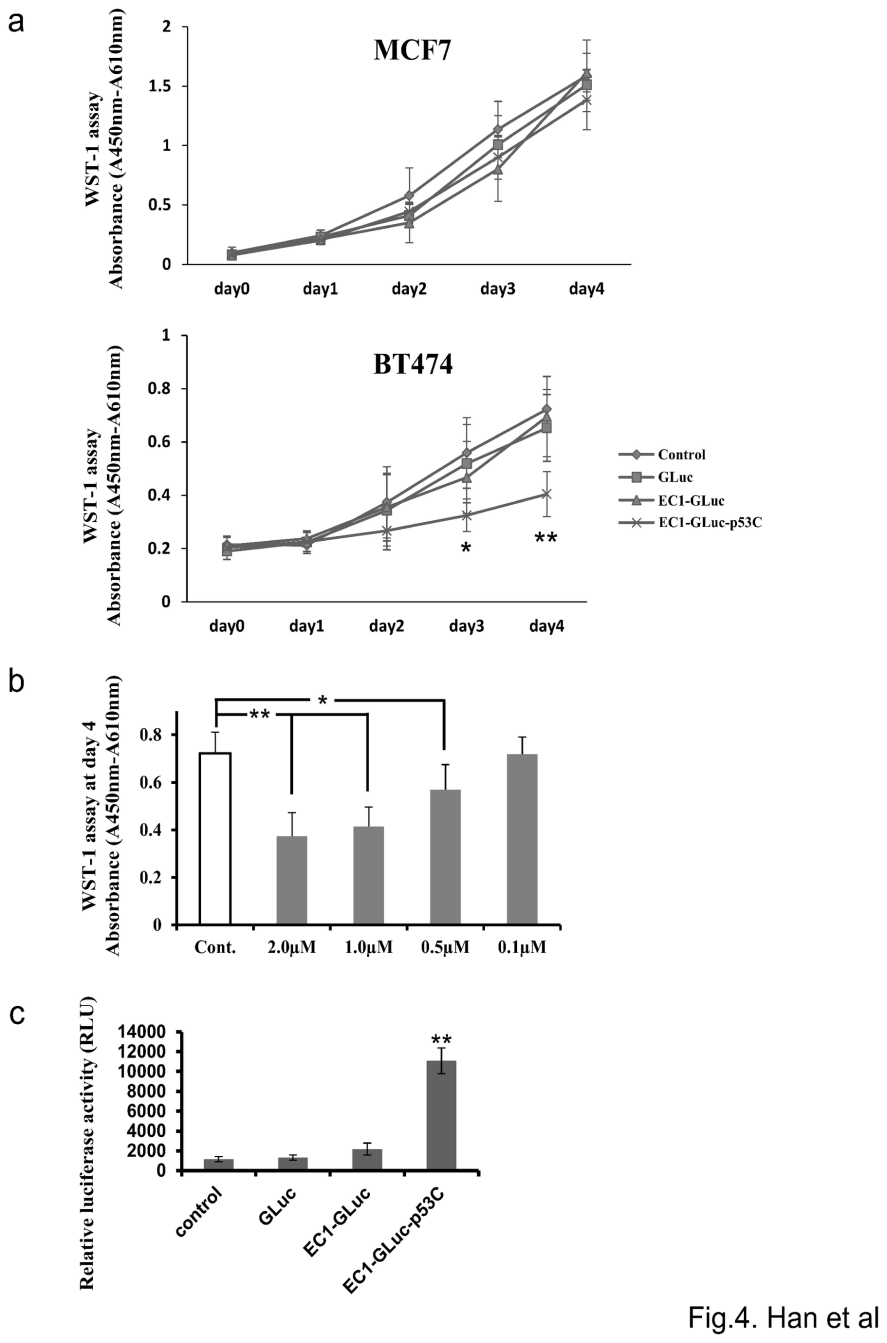
Effect of EC1-GLuc-p53C on proliferation of MCF7 and BT474 cells. a) Time-dependent changes in proliferation of MCF7 (top panel) and BT474 (bottom panel) cells. Cells were supplemented with 1 µM of each recombinant protein or PBS (control) on day 0 and further cultured 96 h (day 4). Cellular proliferation was assessed by the WST-1 assay every 24 h. ♦, PBS (control); ■, GLuc; ▲, EC1-GLuc; ×, EC1-GLuc-p53C. n = 6 each. *p<0.05 and **p<0.01 vs control. b) Dose-dependent effect of EC1-GLuc-p53C on the proliferation of BT474 cells. BT474 cells were treated with EC1-GLuc-p53C at various concentrations or PBS (Cont.), and WST-1 assay was performed on day 4. n = 6 each. *, p < 0.05; **, p < 0.01. c) p53-driven transcriptional activity with the p21^*WAF1*^ luciferace reporter assay. BT474 transfected with the luciferase reporter vector were incubated with 1µM of GLuc, EC1-GLuc, EC1-GLuc-p53C or PBS for 24h. p21^*WAF1*^ luciferase reporter activities in cells were measured with Luciferase Assay System. Data are presented as the mean±S.E.M. *n* = 5 in each group; **P < 0.01.

WST-1 assay was used to evaluate effect of the recombinant proteins on the proliferation of MCF7 and BT474 cells. EC1-GLuc-p53C significantly inhibited the proliferation of BT474 but not MCF7 cells on day 3 and 4 (Figure 4a). The control proteins, GLuc and EC1-GLuc, had no significant effect on the proliferation of either cell line (Figure 4a). Moreover, effect of EC1-GLuc-p53C on the proliferation of BT474 was dose-dependent (Figure 4b). In addition, the results of luciferase reporter Assay for p53-driven Transactivation indicated that p53C peptide was active and reactivated endogenous p53 when internalized in BT474 cells (Figure 4c). Taken together, these results suggest that the inhibitory effect of EC1-GLuc-p53C on proliferation of cancer cells has an ErbB2-targeting property and is due to the efficacy of p53C peptide in the fusion protein.

### EC1-GLuc-p53C targets ErbB2 for bioluminescence imaging *in vivo*

**Figure 5:**
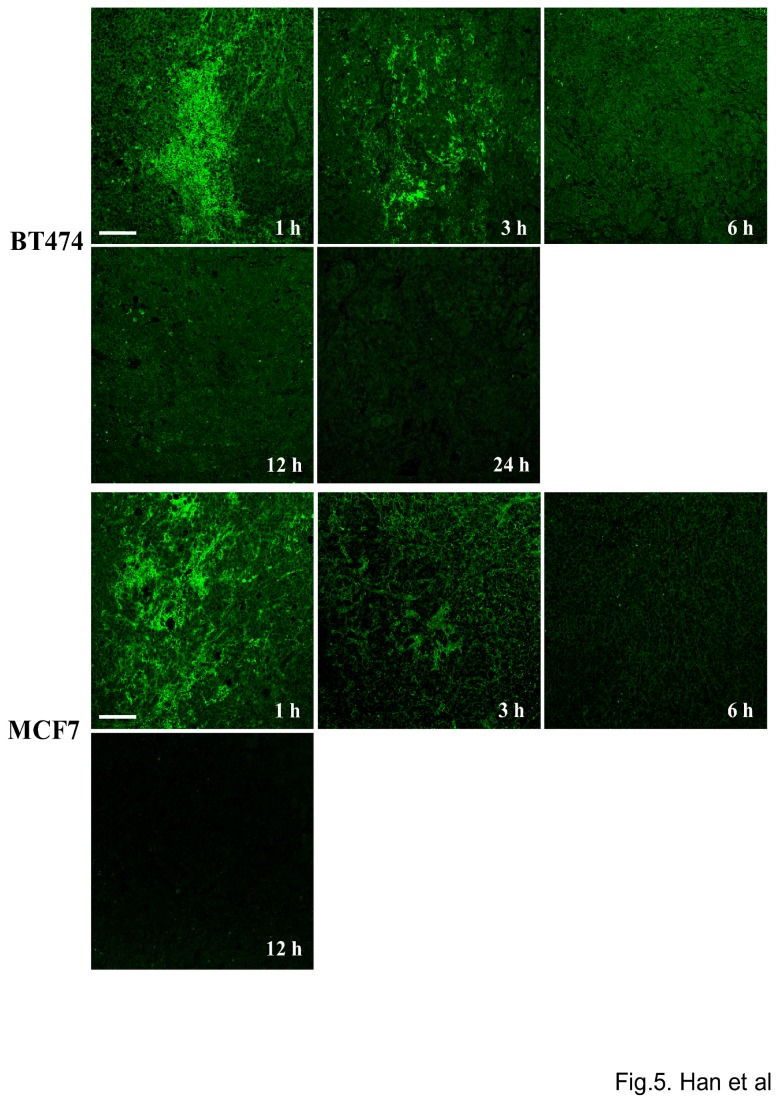
Tumor site retention of EC1-GLuc-p53C after injection. At indicated time points after intratumoral injection of EC1-GLuc-p53C, mice were killed and tumors were excised. Tumors were then immediately fixed with 4% PFA. Paraffin-embedded slices of tumors were prepared at a thickness of 10 µm. Immunofluorescence staining with rabbit anti-*Gaussia* Luciferase serum was used to detect the retention of EC1-GLuc-p53C in tumors. Scale bars = 100 µm. n = 3 in each group.

**Figure 6:**
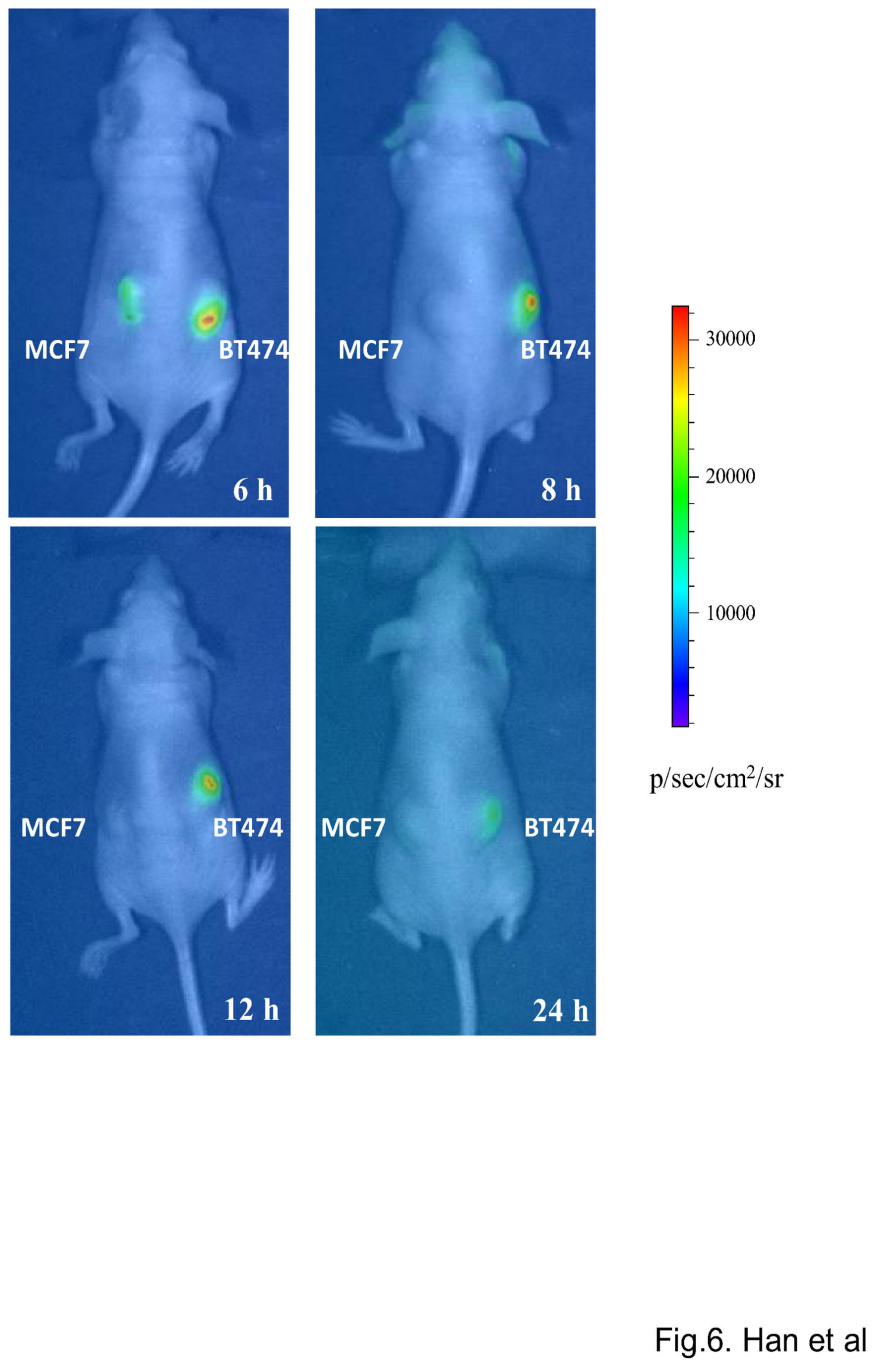
Bioluminescence imaging in living nude mice bearing MCF7 and BT474 tumors. MCF7 and BT474 tumors were established on the left and right flank of nude mice, respectively. At 6, 8, 12 and 24 h after the application of EC1-GLuc-p53C, the mice were imaged immediately after the intravenous injection of CTZ solution (3mg/Kg). The color scale represents p/sec/cm^2^/steradian. n = 3 in each group.

For the experiments *in vivo*, we prepared mice with MCF7 and BT474 xenografted tumors on both flanks. Histological observations of the tumors were made using H&E staining (Figure S2a). High expression of ErbB2 in BT474 tumors was also confirmed by IF staining (Figure S2b). To evaluate the ErbB2-targeting property of EC1-GLuc-p53C *in vivo*, the retention of EC1-GLuc-p53C in tumors after intratumoral injection was examined by IF staining with anti-GLuc antibody. EC1-GLuc-p53C was detectable in BT474 tumors for up to 24 h after the injection. In contrast, EC1-GLuc-p53C was only weakly detected in MCF7 tumors up to 6 h after the injection (Figure 5). The result suggests the retention time of EC1-GLuc-p53C to be much longer in BT474 than MCF7 tumors. Based on the retention time of EC1-GLuc-p53C in tumors, bioluminescent images were acquired 6, 8, 12 and 24 h after the protein injection. Signal was detected in BT474 tumors for up to 24 h, while it was undetectable at MCF7 tumor sites 8 h after the protein injection (Figure 6). These results suggest that the ErbB2-targeting property of EC1-GLuc-p53C facilitates its retention in BT474 tumors and bioluminescence imaging *in vivo*.

### ErbB2-targeted tumor growth inhibition by EC1-GLuc-p53C

**Figure 7:**
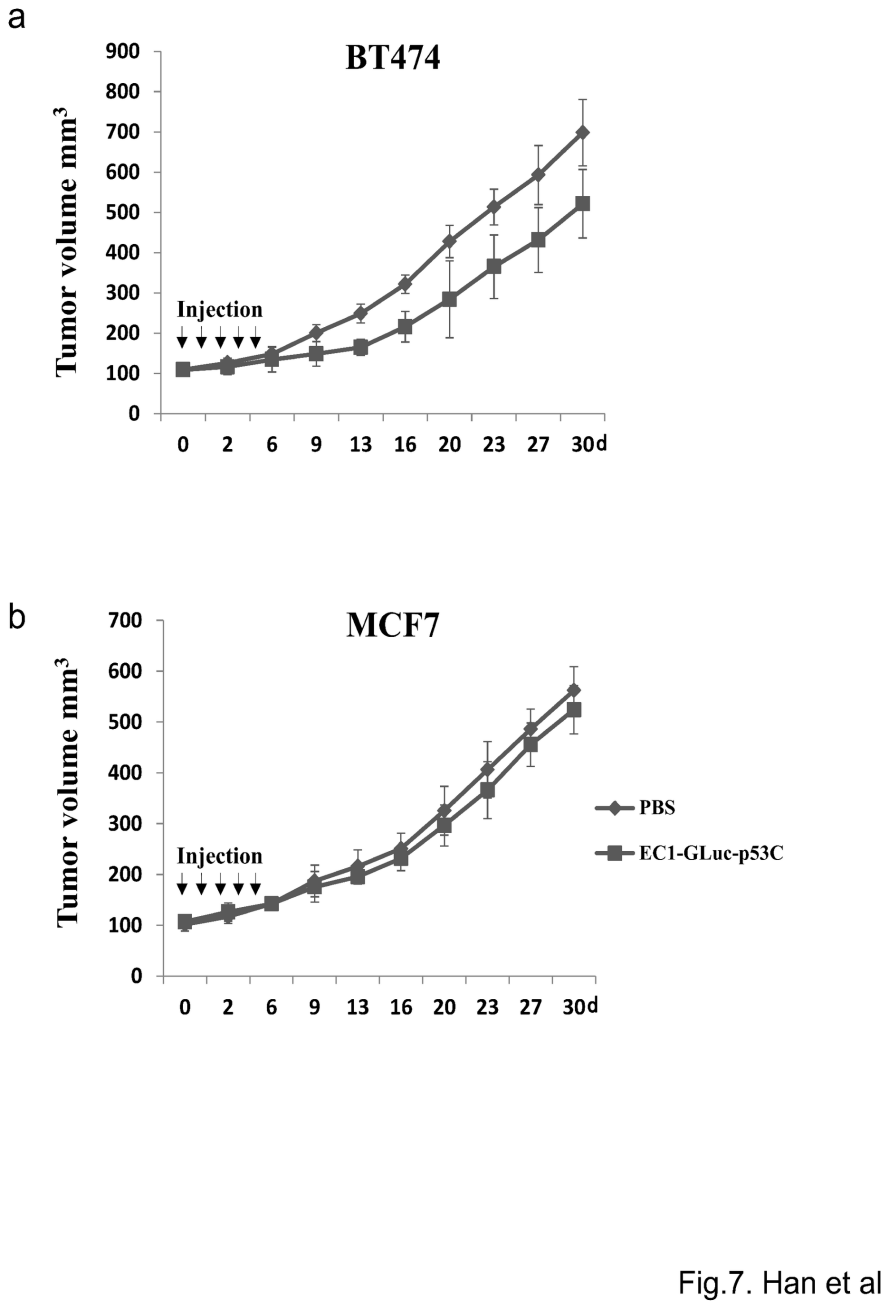
Effect of EC1-GLuc-p53C on tumor growth. 30 µL of EC1-GLuc-p53C (0.5 µg/µL) or the same volume of PBS (control) were injected into MCF7 and BT474 tumors every day for 5 days. The mice were monitored daily and their tumor volume was measured twice a week to evaluate the effect of EC1-GLuc-p53C on tumor growth. n = 9 in each group.

To examine the effect of EC1-GLuc-p53C on tumor growth, mice bearing MCF7 and BT474 tumors with an average volume of ~110mm^3^±10mm^3^ were intratumorally injected with 15 µg of protein every day for 5 days. After treatment, all mice were monitored by measuring tumor volume. The data are plotted in Figure 7. EC1-GLuc-p53C inhibited the growth of BT474 tumors from day 9 after treatment. In contrast, no significant therapeutic effect of the protein was observed in MCF7 tumors. These results suggest that the inhibitory effect of EC1-GLuc-p53C on tumor growth involves the targeting of ErbB2. To clarify the therapeutic effect of EC1-GLuc-p53C on BT474 tumor is due to the efficacy of p53C peptide, BT474 tumors further received treatment with 30 µL of GLuc, EC1-GLuc, EC1-GLuc-p53C (0.5 µg/µL) or the same volume of PBS. Results showed that only EC1-GLuc-p53C significantly inhibited the growth of BT474 tumors (Figure S3). It indicates that the inhibitory effect of EC1-GLuc-p53C on the tumor growth has an ErbB2-targeting property and is derived from the efficacy of p53C peptide in the fusion protein, which is similar to those *in vitro*.

## Discussion

With advances in cancer biology, some surface molecules specifically overexpressed in cancer cells have been identified. Among them, the ErbB/EGFR family is one of the best known. In addition, progress in molecular biology has provided new techniques for developing biomolecules to target molecules of interest for cancer diagnosis and therapy [17]. Previously, we developed an immunoliposome conjugated with anti-EGFR antibody, which successfully targeted glioma cells for delivery of BSH and bioluminescence imaging *in vitro* and *in vivo* [39,43]. In the present study, we designed a novel fusion protein for ErbB2-targeted imaging and cancer therapy. In the fusion protein, EC1 peptide, an artificial ErbB2 ligand, and a p53 reactivating peptide, p53C, were fused to *Gaussia* luciferase. We hypothesized that EC1-GLuc-p53C protein would exhibit the functions of all three components: targeting ErbB2, bioluminescence imaging and anticancer activity. *In vitro* assays showed that the bioluminescent activity of EC1-GLuc-p53C was reduced to approximately 50% of that of GLuc by the N- and C-terminal fusion of the two peptides. However, the bioluminescence of EC1-GLuc-p53C in serum was much more stable than that of GLuc (Figure 2a). At 3 h after incubation in serum, EC1-GLuc-p53C was nearly similar to GLuc in bioluminescence activity (Figure 2b). With the high quantum yield of GLuc [37] and improved stability by peptide fusion, EC1-GLuc-p53C is expected to be suitable for applications *in vivo*.

Unlike other members of the ErbB family, ErbB2 is an orphan receptor without an endogenous ligand. In a phage display study, an artificial peptide EC1 was found to bind to the extracellular domain of ErbB2 [14]. Importantly, the affinity for ErbB2 is retained in biotin-conjugated EC1, EC1-eGFP and EC1-Fc-liposome. In addition, EC1-fused molecules are selectively internalized into ErbB2-overexpressing cancer cells [14-16]. Consistent with previous studies, EC1-GLuc and EC1-GLuc-p53C also selectively targeted ErbB2-overexpressing BT474 cells (Figure 3b and Figure S1). These results suggest the ErbB2-targeting property of EC1 to be retained in our fusion proteins. On the other hand, it was reported that EC1 peptide at 25 and 50 µM inhibited the phosphorylation of ErbB2, and inhibited the growth of ErbB2-overexpressing MCF-7 cells at 1.0 µM [14]. However, we did not observe any significant effect of EC1-GLuc on the proliferation of BT474 cells at 1.0 µM. It is possible that EC1 suffers a loss in bioactivity when fused with GLuc. In contrast, EC1-GLuc-p53C significantly inhibited the proliferation of BT474, and its effect was dose-dependent (Figure 4a, b). Moreover, the p53C peptide in fusion protein was active and reactivated endogenous p53 when internalized in BT474 cells (Figure 4c). It indicates that the inhibitory effect of EC1-GLuc-p53C on cellular proliferation is due to the p53C peptide. Taken together, the *in vitro* results of the present study demonstrated that the three components in the newly designed fusion protein exerted their functions well.

For experiments *in vivo*, 50~75 µg of EC1-GLuc-p53C was injected into tumor-bearing mice via a tail vein, but no significant bioluminescence was detected in tumors at 0.5~12 h after the injection (data not shown). A previous study showed that an N-terminal truncated GLuc, GLΔ15 (20 kDa), was rapidly cleared through the kidneys over 20 min after its injection into mice, while the clearance of a diabody-fused GLΔ15 (90 kDa) was much slower [38]. We supposed that the small size of EC1-GLuc-p53C (25 kDa) might facilitate rapid clearance through the kidneys, and lead to very little accumulation of the fusion protein in tumors for bioluminescence imaging. Therefore, the intravenous injection was replaced with an intratumoral injection for experiments *in vivo*. The tumor site retention of EC1-GLuc-p53C showed that the fusion protein resided in BT474 tumors for up to 24 h after the intratumoral injection, whereas it was only weakly detected in MCF7 tumors up to 6 h (Figure 5), suggesting the longer retention time of EC1-GLuc-p53C in BT474 tumors to be due to its affinity for the ErbB2 receptor. Consistent with the retention of EC1-GLuc-p53C, bioluminescence was specifically detected in BT474 tumors from 8 h after protein injection (Figure 6). In addition, the repeated injection of EC1-GLuc-p53C selectively inhibited the growth of BT474 tumors (Figure 7), while no significant therapeutic effect was observed in BT474 tumors treated with PBS, GLuc or EC1-GLuc (Figure S3). The results suggest that EC1-GLuc-p53C is effective for the bioluminescence imaging and treatment of ErbB2-overexpressing tumors *in vivo*, although further optimization is needed for intravenous administration.

The combination of a diagnostic test and a therapeutic entity is termed theranostics [44,45]. In recent years, theranostics has developed very quickly along with selective targeting strategies. The targeting moieties include proteins (mainly antibodies and their fragments), peptides and some small molecules (folate and flavin mononucleotide) [45]. Peptides are attractive targeting molecules due to their small size, low immunogenicity and ease of manufacture at low costs. In the present research, we used EC1 peptide as a targeting moiety. The fusion protein EC1-GLuc-p53C efficiently targeted ErbB2-overexpressing cancer cells for bioluminescence imaging and therapy *in vitro* and *in vivo*. Thus, our multifunctional EC1 fusion protein should be a promising theranostic reagent for ErbB2-overexpressing tumors, although the development of this technology is still at the early stage. In addition, this system may also provide new theranostic reagents for other cancers with selective targeting moieties.

## Conclusions

The present study showed the utility of a newly constructed protein, EC1-GLuc-p53C, for ErbB2-targeted bioluminescence imaging and cancer therapy *in vitro* and *in vivo*. EC1-GLuc-p53C retained the ErbB2-targeting property, and the enzymatic activity of GLuc in the fusion protein was successfully applied to the bioluminescence imaging of ErbB2-overexpressing BT474 cells *in vitro* and xenografted BT474 tumors *in vivo*; moreover, when internalized into cells, the p53C peptide in the fusion protein was active and exerted anticancer activity to inhibit tumor growth. In conclusion, EC1-GLuc-p53C may be a promising theranostic reagent for ErbB2-overexpressing cancer *in vitro* and *in vivo*.

## Supporting Information

Figure S1 **Bioluminescence imaging of cells treated with EC1-GLuc and GLuc *in vitro*.** After incubation with 1 µM of EC-GLuc (c-d and g-h) or GLuc (a-b and e-f), MCF7 (a-d) and BT474 (e-h) cells were washed with culture medium without serum. Images were acquired with a bioluminescence microscope immediately after the addition of 1 µg/mL CTZ. Bars = 100 µm.
(TIF)

Figure S2 **Confirmation of the established MCF7 and BT474 xenografted tumors.** a) Histological observations of xenografted tumors were made using H&E staining. Bar = 1.0 mm. b) Expression of ErbB2 in MCF7 and BT474 tumors. Paraffin-embedded slices of tumors were subjected to IF staining with rabbit anti-ErbB2 antibody and Hoechst 33248 for nuclear staining. Bars = 100 µm. n = 3 in each group.
(TIF)

Figure S3 **Therapeutic Effect of EC1-GLuc-p53C on tumor growth is derived from the efficacy of p53C.** 30 µL of GLuc, EC1-GLuc, EC1-GLuc-p53C (0.5 µg/µL) or the same volume of PBS were injected into BT474 tumors every day for 5 days. Tumor volume was measured twice a week to monitor tumor growth. n = 7 in each group.
(TIF)
