# Supplementary figures and images for: Theranostic Protein Targeting ErbB2 for Bioluminescence Imaging and Therapy for Cancer
Source: PLoS One. 2013 Sep 17;8(9):e75288. doi: 10.1371/journal.pone.0075288 (PMC3775930; doi:10.1371/journal.pone.0075288)

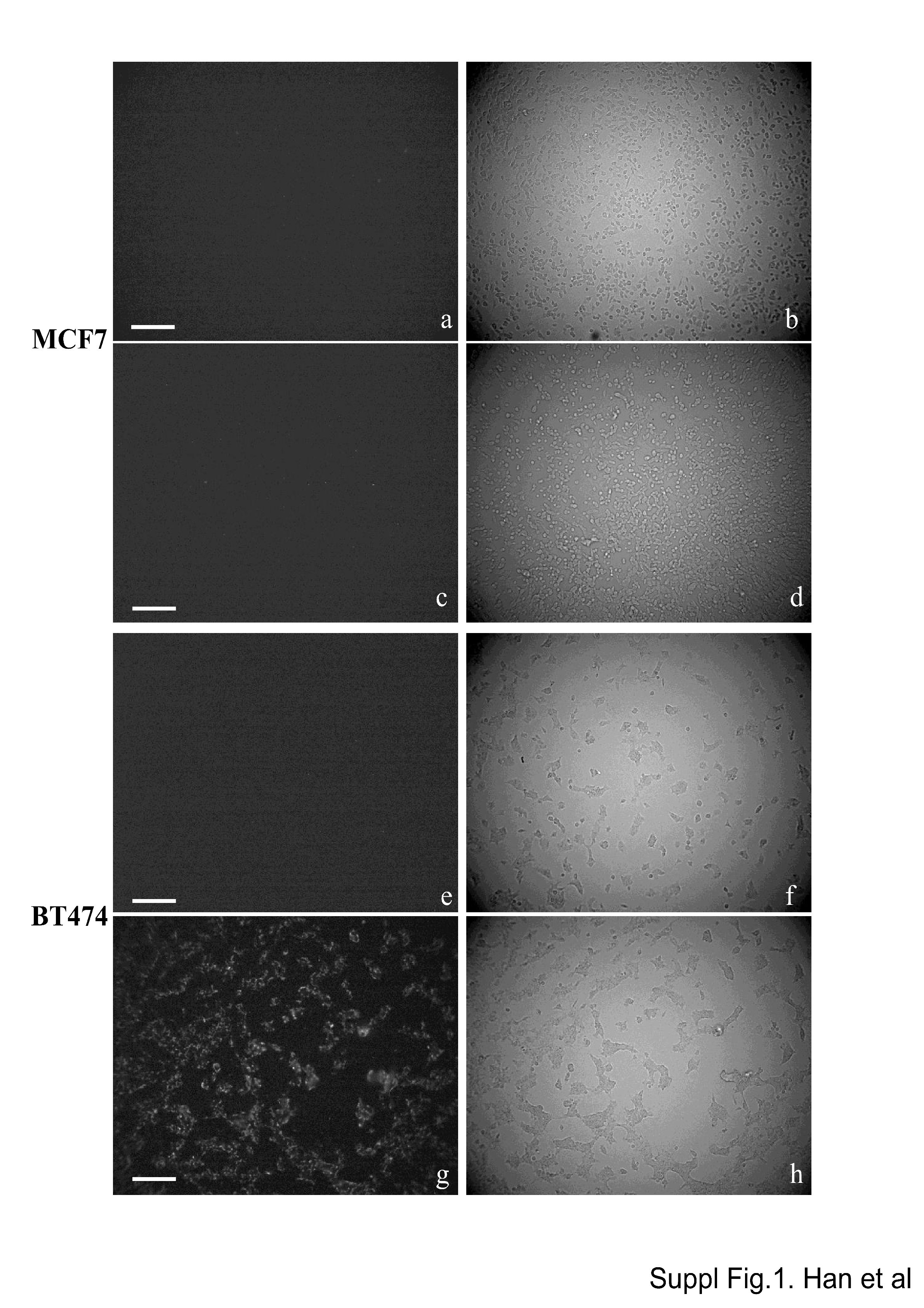

Supplement: Figure S1 — Bioluminescence imaging of cells treated with EC1-GLuc and GLuc in vitro. After incubation with 1 µM of EC-GLuc (c-d and g-h) or GLuc (a-b and e-f), MCF7 (a-d) and BT474 (e-h) cells were washed with culture medium without serum. Images were acquired with a bioluminescence microscope immediately after the addition of 1 µg/mL CTZ. Bars = 100 µm. (TIF) [file pone.0075288.s001.tif]

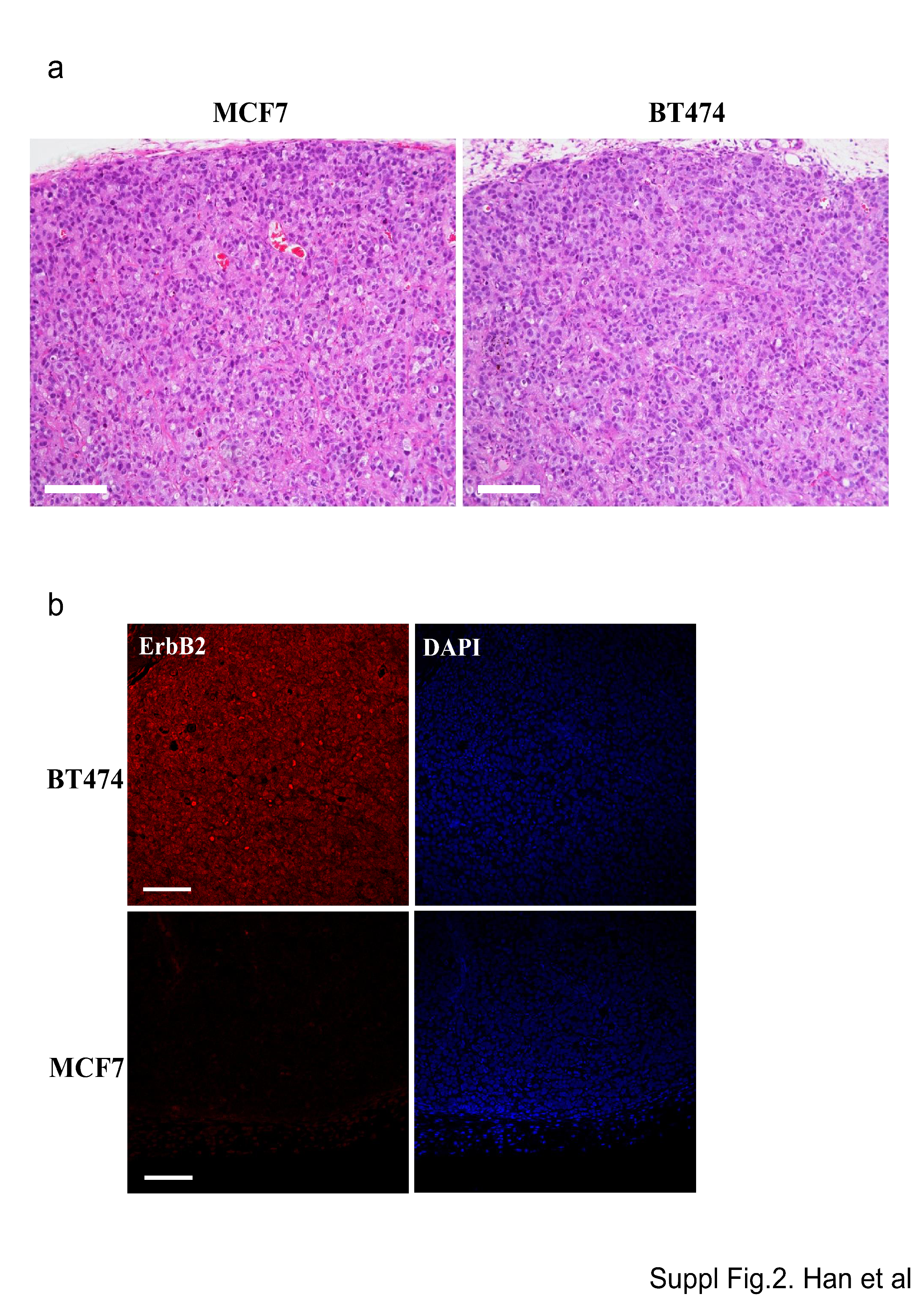

Supplement: Figure S2 — Confirmation of the established MCF7 and BT474 xenografted tumors. a) Histological observations of xenografted tumors were made using H&E staining. Bar = 1.0 mm. b) Expression of ErbB2 in MCF7 and BT474 tumors. Paraffin-embedded slices of tumors were subjected to IF staining with rabbit anti-ErbB2 antibody and Hoechst 33248 for nuclear staining. Bars = 100 µm. n = 3 in each group. (TIF) [file pone.0075288.s002.tif]

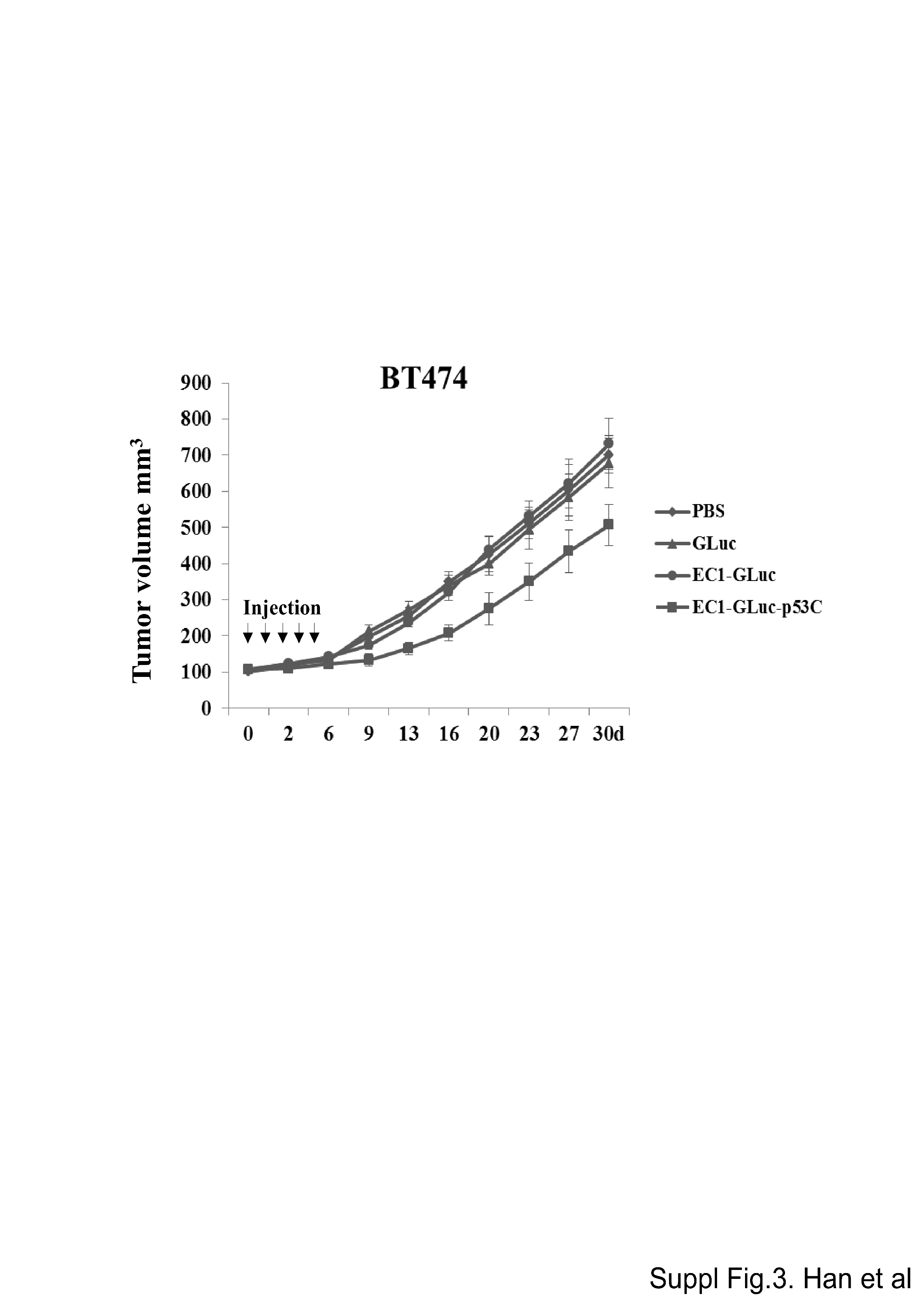

Supplement: Figure S3 — Therapeutic Effect of EC1-GLuc-p53C on tumor growth is derived from the efficacy of p53C. 30 µL of GLuc, EC1-GLuc, EC1-GLuc-p53C (0.5 µg/µL) or the same volume of PBS were injected into BT474 tumors every day for 5 days. Tumor volume was measured twice a week to monitor tumor growth. n = 7 in each group. (TIF) [file pone.0075288.s003.tif]
